# The Mitochondrial Genome of *Baylisascaris procyonis*

**DOI:** 10.1371/journal.pone.0027066

**Published:** 2011-10-28

**Authors:** Yue Xie, Zhihe Zhang, Lili Niu, Qiang Wang, Chengdong Wang, Jingchao Lan, Jiabo Deng, Yan Fu, Huaming Nie, Ning Yan, Deying Yang, Guiying Hao, Xiaobin Gu, Shuxian Wang, Xuerong Peng, Guangyou Yang

**Affiliations:** 1 Department of Parasitology, College of Veterinary Medicine, Sichuan Agricultural University, Ya'an, China; 2 The Sichuan Key Laboratory for Conservation Biology on Endangered Wildlife – Developing Toward a State Key Laboratory for China, Chengdu Research Base of Giant Panda Breeding, Chengdu, Sichuan, China; 3 Chengdu Zoological Garden, Chengdu, Sichuan, China; 4 Department of Chemistry, College of Life and Basic Science, Sichuan Agricultural University, Ya'an, China; University of Georgia, United States of America

## Abstract

**Background:**

*Baylisascaris procyonis* (Nematoda: Ascaridida), an intestinal nematode of raccoons, is emerging as an important helminthic zoonosis due to serious or fatal larval migrans in animals and humans. Despite its significant veterinary and public health impact, the epidemiology, molecular ecology and population genetics of this parasite remain largely unexplored. Mitochondrial (mt) genomes can provide a foundation for investigations in these areas and assist in the diagnosis and control of *B. procyonis*. In this study, the first complete mt genome sequence of *B. procyonis* was determined using a polymerase chain reaction (PCR)-based primer-walking strategy.

**Methodology/Principal Findings:**

The circular mt genome (14781 bp) of *B. procyonis* contained 12 protein-coding, 22 transfer RNA and 2 ribosomal RNA genes congruent with other chromadorean nematodes. Interestingly, the *B. procyonis* mtDNA featured an extremely long AT-rich region (1375 bp) and a high number of intergenic spacers (17), making it unique compared with other secernentean nematodes characterized to date. Additionally, the entire genome displayed notable levels of AT skew and GC skew. Based on pairwise comparisons and sliding window analysis of mt genes among the available 11 Ascaridida mtDNAs, new primer pairs were designed to amplify specific short fragments of the genes *cytb* (548 bp fragment) and *rrn*L (200 bp fragment) in the *B. procyonis* mtDNA, and tested as possible alternatives to existing mt molecular beacons for Ascaridida. Finally, phylogenetic analysis of mtDNAs provided novel estimates of the interrelationships of *Baylisasaris* and Ascaridida.

**Conclusions/Significance:**

The complete mt genome sequence of *B. procyonis* sequenced here should contribute to molecular diagnostic methods, epidemiological investigations and ecological studies of *B. procyonis* and other related ascaridoids. The information will be important in refining the phylogenetic relationships within the order Ascaridida and enriching the resource of markers for systematic, population genetic and evolutionary biological studies of parasitic nematodes of socio-economic importance.

## Introduction

*Baylisascaris procyonis*, a ubiquitous helminth parasite of raccoons (*Procyon lotor*), is increasingly being recognized as an emerging public health concern in North America, Europe and parts of Asia [1], [2]. *B. procyonis* is the most common cause of clinical visceral (VLM), ocular (OLM) and neural larva (NLM) migrans in various species of birds and mammals, including humans [3]. Humans, as accidental intermediate hosts, become infected by the accidental consumption of infective *B. procyonis* eggs from the environment or articles contaminated with raccoon faeces [4]. Human infection with *B. procyonis* typically results in fatality or long-term neurological sequelae [5]–[10]. Clinical manifestations include eosinophilic encephalitis, ocular disease and eosinophilic cardiac pseudotumor. Fifteen recognized human cases of *B. procyonis* NLM, six of them fatal and predominantly involving children, were reported by Murray and Kazacos (2004). More than 12 additional unpublished cases of infection are also known (K.R. Kazacos, personal observation) [2], [11]. Epidemiological studies suggest that pica or geophagia and exposure to infected raccoons or environments contaminated with their faeces are the most important risk factors for human infection with *B. procyonis*. Current diagnosis of this parasitic infection is typically based on morphological examination. However, morphological characteristics can often be unrecognized even by experienced microscopists, and mistaken identification, particularly of helminth larvae, is not uncommon [12]. Moreover, the diagnosis becomes more difficult when identification and differentiation of eggs or larval are performed among a number of possible environmental cross-contaminating eggs of other parasites, including morphologically similar *Baylisascaris* spp. [2], and of possible contaminating nematode larvae, including those of *Toxocara canis*, *Toxocara cati*, *Toxascaris leonina*, *Ascaris lumbricoides*, and species of *Angiostrongylus* and *Ancylostoma* [12], [13]. Therefore, obtaining a more efficient and reliable way to identify and differentiate *B. procyonis* eggs or larvae has become crucial for clinical diagnosis, epidemiological investigation and laboratory tests, and achieving this goal is foreseeable only through utilization of molecular methodologies.

Recently, mitochondrial (mt) genomics have received increased attention, and mt DNA is regarded as an important and efficient source of genetic markers, being widely used for species-specific identification and differentiation of many zoonotic nematodes. Sequences of the mt cytochrome-oxydase I (*cox1*) and NADH dehydrogenase subunit 4 (*nad4*) genes have been used to identify and differentiate hookworm species [14] and other Strongylida [15], respectively. Additionally, the cytochrome-oxydase II (*cox2*) gene has also proven useful as a genetic marker for differentiation of species among *T. canis*, *T. cati*, *T. leonina*, *Ascaris suum* and *B. procyonis* [2], [16]. Surprisingly, based on the *cox2* gene, *B. procyonis* and *Baylisascaris columnaris* cannot be distinguished between each other because of the high nucleotide sequence similarity [2]. Similar problems may be further exacerbated in attempts to perform species-specific differentiation between *B. procyonis* and other *Baylisascaris* spp., thus limiting the ability to accurately identify and to assess genetic variability in *B. procyonis* populations, which would be problematic for studies of its epidemiology, diagnosis and control. Compared to the use of partial mt genes, a complete mt genomic dataset would be especially powerful for displaying sufficient interspecies sequence variability and describing species specificity [15], [17]. Moreover, mt genomes contain useful genetic markers for studying the genetic structure within and among *Baylisascaris* spp., due to mutation rates which are proposed to be more rapid than nuclear genes, and presumed lack of recombination and maternal inheritance [15], [18]–[22]. However, no complete information on the mt genome of *B. procyonis* was previously available.

Herein, we first report the complete nucleotide sequence of the mt genome from a representative *B. procyonis* from China and compare the sequence and genome organization with the three other available complete mt genomes from the congeneric *Baylisascaris schroederi*, *Baylisascaris ailuri* and *Baylisascaris transfuga* [23], as well as the sequences from related nematodes in the same order. Based on comparative mitogenomics, whether mt gene fragments currently utilized as genetic markers (such as *cox2*) offer the best regions for characterization or species identification and recognition is discussed. Additionally, new PCR primer pairs designed to amplify short fragments of mtDNA for *B. procyonis* were developed with the aim of providing the ability to differentiate between *B. procyonis* and other species of ascaridoids, including morphologically similar *Baylisascaris* spp. Finally, the phylogenetic relationships of the species *B. procyonis* within the genus *Baylisasaris* and of the genus *Baylisascaris* within the order Ascaridida were also investigated by the construction of phylogenetic trees (NJ, MP and ML) using the protein-coding amino acid sequence dataset.

## Results and Discussion

### Main features of the mt genome of *B. procyonis*

**Figure 1:**
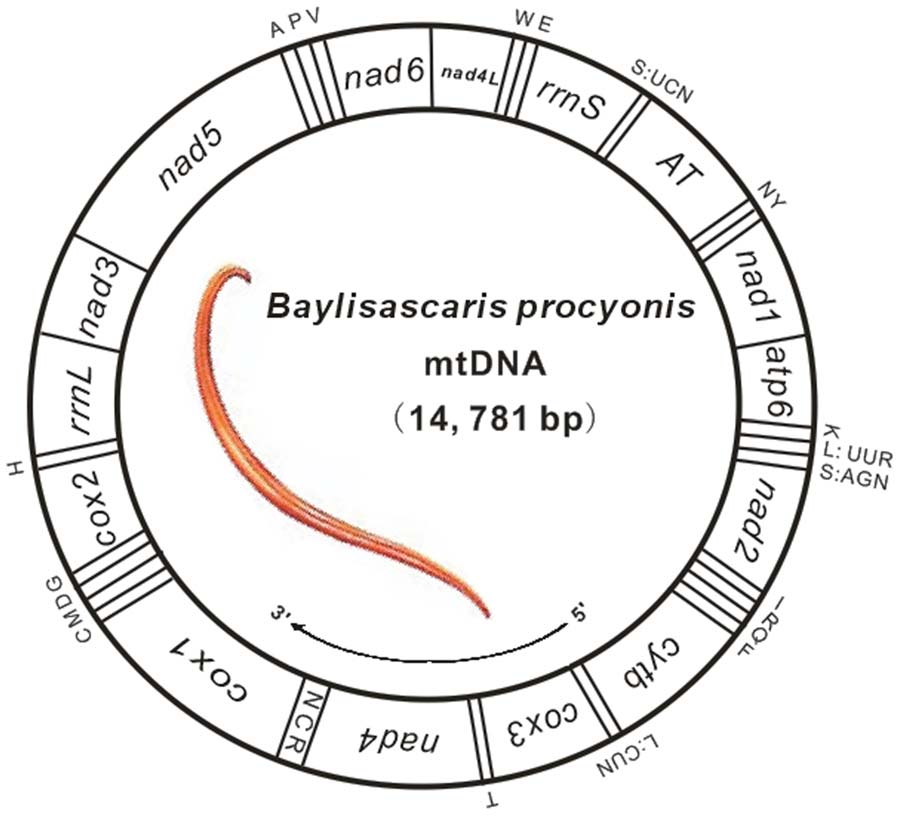
Graphical representation of *B. procyonis* mt genome. Gene abbreviations are as follows: *atp6*, ATP synthase subunits 6; *cox1-3*, cytochrome oxidase c subunits 1–3; *cytb*, cytochrome b; *nad1-nad6* and *nad4*L, NADH dehydrogenase subunits 1–6 and 4L; *rrn*L and *rrn*S, ribosomal RNAs. Transfer RNA genes are indicated by one letter symbol according to the IPUC-IUB single-letter amino acid codes. The two leucine and the two serine tRNA genes are differentiated by their respective anti-codons. AT denotes the AT-rich region. The direction of transcription is indicated by an arrow (5′ to 3′).

The complete mt genome of *B. procyonis* was 14781 bp in size (GenBank accession No. JF951366) and encoded 36 genes, including 12 protein-coding genes (1 subunit of the ATP synthase, *atp6*; 3 subunits of cytochrome c oxidase, *cox1-3*; 1 subunit of cytochrome c-ubiquinol oxidoreductase, *cytb*; and 7 subunits of NADH dehydrogenase, *nad1-6* and *nad4*L), 22 transfer RNA (*trn*) genes (two coding for leucine and two coding for serine) and the small and large subunit ribosomal RNAs (*rrn*S and *rrn*L) (Figure 1; Table S1). As with other chromadorean nematode mtDNAs sequenced thus far, the *B. procyonis* mt genome also lacked the gene encoding *atp8*. All genes were distributed on the same strand and transcribed in the same direction (5′ to 3′), typical for other nematodes reported to date (except for *Trichinella spiralis* and *Xiphinema americanum*) (Figure 1) [24], [25]. Gene order for *B. procyonis* mtDNA followed the GA7 arrangement [26], with the exception of the relative positions of the AT-rich region and the number of non-coding regions (NCRs). A similar gene arrangement was described previously for members of the orders Ascaridida and Strongylida as well as for the free-living nematode *Caenorhabditis elegans* [23], [27]–[33]. Only one long unassigned region was present between the genes *rrn*S and *nad1*, flanked at the 5′ end by the gene *trn*S (UCN) and at the 3′ end by the genes *trn*N and *trn*Y, and it was deemed homologous to the AT-rich region (also known as the control region) by analysis of positional homology, general structure and base content. In addition, the *B. procyonis* mtDNA contained 17 intergenic spacers ranging in length from 1 to 118 bp (211 bp in total) including the NCR region (Table S1), which was the highest number of intergenic spacers identified in a nematode mt genome thus far [23]. There were only two overlaps found between the genes, with one (1 bp) between *cox1* and *trn*C and another (3 bp) between *trn*F and *cytb* (see Table S1).

### Base composition and codon usage

The overall base composition (coding strand) for the mt genome sequence of *B. procyonis* was as follows: A = 22.0%, C = 8.1%, G = 21.4%, T = 48.5%. The A+T bases comprised 68.6% of the protein-coding genes, 72.0% of the rRNAs, 69.6% of the tRNAs and 82.8% of the AT-rich region, giving a total A+T content of 70.5% in this mt genome. This figure was slightly higher than that in the congeneric species *B. ailuri* (69.5%), *B. transfuga* (69.4%) and *B. schroederi* (68.6%), and it was well within the range of the AT contents reported for other nematode species in the same order (68.3–72.0%) (Table 1). In general, the AT and GC skews on the two complementary DNA strands for each mtDNA are regarded as a measure of the compositional asymmetry [34]. For the entire *B. procyonis* mtDNA, the AT and GC skews for the coding strand were −0.376 and 0.448, respectively, which were significant compared with those of other nematodes characterized to date (AT skews ranging from −0.384 to −0.353, and GC skews from 0.320 to 0.457). A similar trend was also observed in the protein-coding genes. The nucleotide frequency of the protein-coding genes was observed to be in the order T > G > A > C, which excessively favored T (50.8%) and was skewed against C (8.31%). This nucleotide bias would have an appreciable effect on both the codon usage pattern and the relative synonymous codon usage (RSCU). Indeed, the protein-coding genes of *B. procyonis* were biased towards codons with many T residues [e.g., 13.9% were TTT (phenylalanine)] over those with many C residues [e.g., <0.1% were TCC (serine) or CTC (leucine)] (see Table S2). This phenomenon could be explained by synonymous codon usage bias. Generally codon bias is proposed to be highest in gene regions of functional significance and believed to be important for maximizing translation efficiency [35], [36]. Interestingly, similar nucleotide bias was also reflected in the choices of initiation and termination codons. The most frequently used start codon for *B. procyonis* was TTG (6 of 12 protein-coding genes; *cox1*-*2*, *nad1*, *nad3*-*4* and *nad6*) followed by GTG (three genes; *nad2*, *cytb* and *cox3*), and ATT (*nad4*L and *nad5*) and ATA (*atp6*) were also used as initiation codons (Table S1). Ten of the 12 protein-coding genes were predicted to use TAG (*atp6*, *cytb*, *cox1*-*3*, *nad1*, *nad4* and *nad6*) or TAA (*nad3* and *nad4*L) as the termination codons, while the remaining genes (*nad2* and *nad5*) were deduced to end with an incomplete codon T.

**Table 1:** Size and nucleotide composition of different genomic regions in 11 ascarids reported within Ascaridida.

Species	mtDNA		PCGs^b^		rRNAs		tRNAs		AT-region		References
	Size a	AT%	Size a	AT%	Size a	AT%	Size a	AT%	Size a	AT%	
***Anisakis simplex***	13916	71.2	10274	69.5	1656	74.3	1208	72.4	515	87.2	Kim *et al.* (2006)
***Ascaris suum***	14284	72.0	10397	70.5	1661	74.7	1252	71.0	886	84.6	Okimoto *et al.* (1992)
***Baylisascaris ailuri***	14657	69.5	10287	67.9	1657	69.5	1241	67.0	1282	82.0	Xie *et al.* (2011)
***Baylisascaris procyonis***	14781	70.5	10289	68.6	1664	72.0	1246	69.6	1375	82.8	This study
***Baylisascaris schroederi***	14778	68.6	10290	67.1	1657	69.8	1241	67.3	1406	78.9	Xie *et al.* (2011)
***Baylisascaris transfuga***	14898	69.4	10290	67.6	1658	69.7	1244	67.5	1516	82.3	Xie *et al.* (2011)
***Contracaecum rudolphii*** ** B**	14022	70.4	10281	69.0	1650	72.3	1256	70.6	588	89.1	Unpublished
***Toxocara canis***	14163	68.3	10294	67.3	1617	69.6	1222	69.3	828	78.1	Jex *et al.* (2008)
***Toxocara canis***	14322	68.6	10308	67.2	1655	69.8	1251	68.5	975	79.5	Li *et al.* (2008)
***Toxocara cati***	14029	69.9	10284	68.8	1651	71.5	1248	70.2	711	81.3	Li *et al.* (2008)
***Toxocara malaysiensis***	14266	68.9	10297	67.8	1651	68.5	1252	70.3	936	78.4	Li *et al.* (2008)

a In base pairs.

b All protein-coding genes were taken into account.

**Figure 2:**
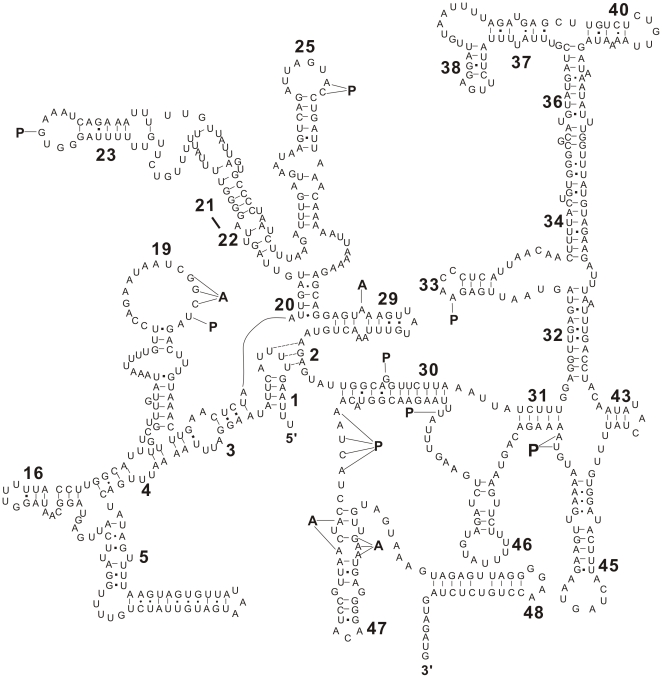
Predicted secondary structure for the small ribosomal RNA gene subunit (*rrn*S) in *B. procyonis* mtDNA. Base-pairing is indicated as follows: Watson-Crick pairs by lines, wobble GU pairs by large dots and other non-canonical pairs by small dots. Conserved secondary structure elements are denoted by bold numbers (1–48) [29]. Binding sites for the amino-acyl *trn* (A) or peptidyl-transferase (P) [54] are indicated by lines.

**Figure 3:**
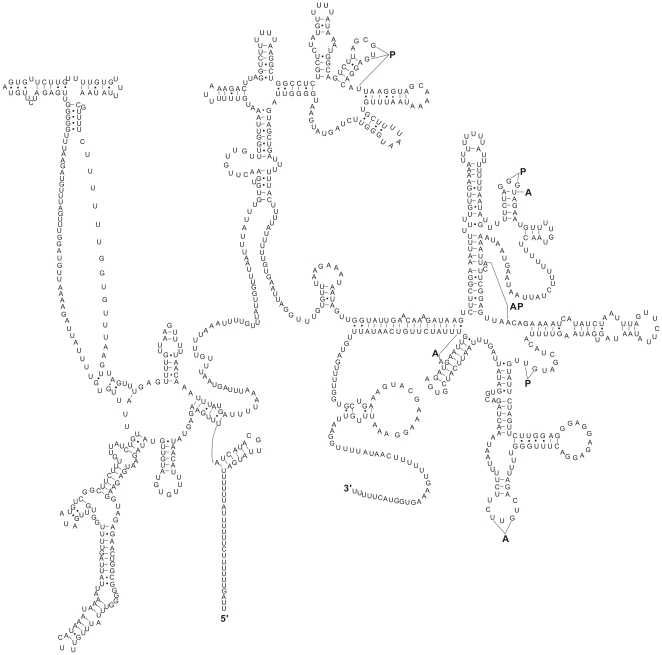
Predicted secondary structure for the large ribosomal RNA gene subunit (*rrn*L) in *B. procyonis* mtDNA. Symbols for base-pairings are as in Figure 2. Binding sites for the amino-acyl *trn* (A), peptidyl-transferase (P) or both (AP) [54] are indicated by lines.

Twenty-two tRNAs were predicted in *B. procyonis* mtDNA, ranging from 51 to 62 bp in size, and all anti-codon sequences were the same as in other nematodes examined [23], [28] (see Table S1). Their secondary structures were similar to those of all other secernentean nematodes studied to date [23], [27]–[33], [37]–[39], but they were distinctly different from the conventional cloverleaf-like structures described in other metazoan mtDNAs (Figure S1). The lengths of *B. procyonis rrn*S and *rrn*L were 700 bp and 964 bp, respectively, and the corresponding secondary structures are shown in Figure 2 (*rrn*S) and Figure 3 (*rrn*L). However, it seemed that the relatively high AT content displayed in *B. procyonis* mtDNA was conspicuous for the tRNAs and rRNAs genes, compared with those of the congeneric species. The AT-contents of the rRNAs sequence were 2.5%, 2.2% and 2.3% greater than that of *B. ailuri* (69.5%), *B. schroederi* (69.7%) and *B. transfuga* (69.8%), respectively. Likewise, the AT contents of the tRNAs sequence were 2.6%, 2.3% and 2.1% more than those found in *B. ailuri* (67.0%), *B. schroederi* (67.3%) and *B. transfuga* (67.5%), respectively (Table 1) [23]. Interestingly, this level of AT content of the *B. procyonis* mt tRNAs and rRNAs genes does not influence their secondary structures. As shown in Figures S1, 2 and 3, the secondary structures of tRNAs and rRNAs in *B. procyonis* were similar with those exhibited in the congeneric *B. ailuri*, *B. transfuga* and *B. schroederi* as well as other nematodes described in Ascaridida to date [23], [27], [28], [30], [33].

The length of the AT-rich region was 1375 bp in the *B. procyonis* mtDNA (Table S1 or Figure 1), and its predicted complex stem-loop structures are shown in Figure S2. This region along with its counterpart in *B. schroederi* (1406 bp), *B. ailuri* (1282 bp) and *B. transfuga* (1516 bp) represented the longest studied thus far among most other secernentean nematodes [23]. In secondary structure analysis, it appeared that there was an additional stem-loop structure at the end of the AT-rich region in *B. procyonis* compared with that of *B. ailuri*, while some stems or loops were missing compared with that of *B. transfuga* and *B. schroederi* (not shown). These differences may relate to the AT-rich region as being the most variable portion of the genome both in terms of length and nucleotide sequence. However, many similar stem-loop structures found in these four *Baylisascaris* species implied that they may be conserved and function in regulation of transcription and control of DNA replication [40]. Additionally, the AT-rich region of the *B. procyonis* mtDNA contained 31 regions with varying numbers of the dinucleotide (TA) repeat (n = 3 to 21) within a total of 344 bp. Similar multiple TA repeats have been described in the AT-rich region of the mt genomes for other Ascaridida and Strongylida species [23], [27]–[33]. Currently the function or role of these AT repeats remains unclear. Other repetitive elements, such as CR1-CR6 identified in the *C. elegans* AT-rich region [27] were not found in *B. procyonis*.

### Levels of variability and informativeness within and between Ascaridida mtDNAs

The comparison of protein-coding and rRNA genes in *B. procyonis* mtDNA with those of ten other published Ascaridida nematodes [*B. ailuri*, *B. transfuga*, *B. schroederi* and *A. suum* (Ascarididae family); *Anisakis simplex* and *Contracaecum rudolphii* B (Anisakidae family); *T. canis*, *T. cati* and *Toxocara malaysiensis* (Toxocaridae family)] is shown in Table S3. The deduced length of the 12 protein-coding genes were consistent with those of the congeneric *B. ailuri*, *B. transfuga* and *B. schroederi*, except for the *cox1* (which was one amino acid longer than that of *B. ailuri*) and *nad2* (which was one nucleotide shorter than that of *B. transfuga* and *B. schroederi*) genes, and along with two rRNA genes were in the size range of those of the other seven nematode mtDNAs. The nucleotide and amino acid sequences similarities for each of the 12 mt proteins of *B. procyonis* ranged from 85.9–92.2% and 83.9–98.3%, respectively, between *B. procyonis* and the congeneric *B. ailuri*, *B. transfuga* and *B. schroederi*; and from 82.5–92.1% and 82.3–97.9%, respectively, between *B. procyonis* and *A. suum*. The nucleotide and amino acid sequences similarities between *B. procyonis* and each species of Toxocaridae were 77.6–89.1% and 74.0–95.4%, respectively; and 73.8–85.9% and 71.5–93.1%, respectively, between *B. procyonis* and each species of Anisakidae. Based on the sequence similarities, the most conserved protein-coding genes among the 11 species (including *B. procyonis*) were *cox1* and *cox2*, while the least conserved were *cytb* and *nad4*. For the genes *rrn*S and *rrn*L, the highest nucleotide similarities were observed between those of *B. procyonis* and the congeneric *B. ailuri*, *B. transfuga*, *B. schroederi* and *A. suum*, with the percent identities being above 91.0% and 85.9%, respectively, followed by the similarities between *B. procyonis* and individual species representing the Toxocaridae and Anisakidae families, with the percent identities ranging from 78.1–81.9% and 75.9–79.2%, respectively (Table S3). In addition, the nucleotide sequence of the AT-region in the *B. procyonis* mtDNA appeared to share low similarity (all values <60%) with that of the ten described Ascaridida species, including the congeneric *B. ailuri*, *B. transfuga* and *B. schroederi* (not shown). Combined, these results from pairwise comparisons of nucleotide and amino acid sequences from the protein-coding genes as well as the nucleotide sequences of the rRNA genes suggested that *B. procyonis* mtDNA most closely resemble those of members of the Ascarididae family, followed by members of the Toxocaridae and Anisakidae families.

**Figure 4:**
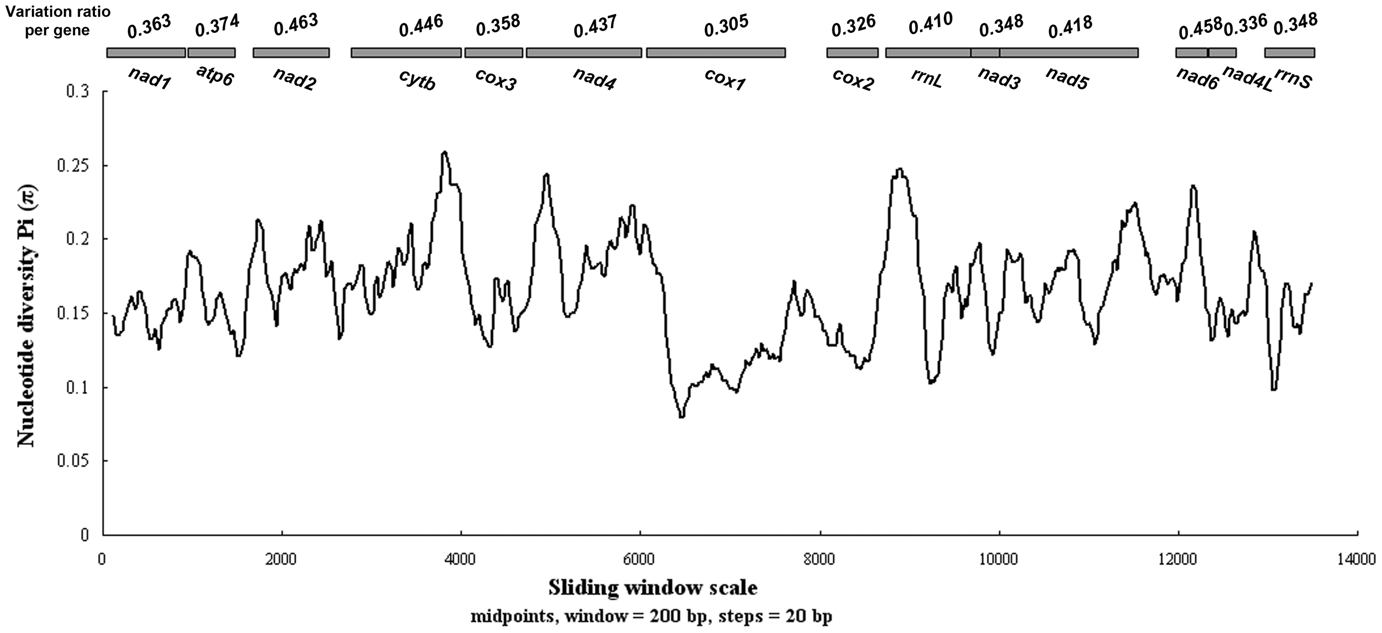
Sliding window analysis of the alignment of complete mtDNAs of 11 species of Ascaridida. The black line shows the value of nucleotide diversity Pi (π) in a sliding window analysis of window size 200 bp with step size 20, and the value is inserted at its mid-point. Gene boundaries are indicated with a variation ratio per gene.

Sliding window analysis of the complete nucleotide alignment of 11 available Ascaridida mtDNAs provided an indication of nucleotide diversity Pi (π) within and between mt genes (see Figure 4). In the curve, the nucleotide variation within and between mt genes among the aligned Ascaridida genomes was intuitively displayed for any given window of 200 bp and steps of 20 bp, with the Pi (π) ranging from 0.075 to 0.262. Coupled with computation of the number of variable positions per unit length of gene, the sliding window showed that the genes with low sequence variability included *cox1* (0.305), *cox2* (0.326), *nad4*L (0.336), *nad3* (0.348) and *rrn*S (0.348), while the genes with high sequence variability included *nad2* (0.463), *nad6* (0.458), *cytb* (0.446), *nad4* (0.437), *nad5* (0.418) and *rrn*L (0.410). Interestingly, amongst the genes with high sequence variability, the genes with pronounced peaks and troughs of Pi (π) appeared to possess higher sequence variability than others, such as *nad2*, *cytb*, *nad4*, *nad5* and *rrn*L (see Figure 4). Based on these results, it seemed that *cox1* and *cox2* were still the most conserved protein-coding genes, and *cytb* and *nad4* were still within the least conserved ones. These observations were remarkably consistent with the findings from pairwise comparisons made among the nucleotide and amino acid sequences from the protein-coding genes in *B. procyonis* mtDNA with those of the other ten published Ascaridida nematodes. These results further suggested that there are still a considerable number of alternative genes (aside from the *cox2* gene [2]) to be determined as new genetic markers for phylogenetics, population genetics and diagnostics. Current mt genes used as molecular targets for PCR assays based approaches for detection/diagnostics in the order Ascaridida include *cox2* and *cytb* [2], [41], and *cox2* is also targeted for development of a probe-based (using a molecular beacon) real-time PCR for diagnosis [16]. Although relatively easy to amplify routinely, based on pairwise comparison and sliding window analysis of mt genes among the available 11 Ascaridida mtDNAs, *cox2* is among the slowest evolving and least variable genes available in *B. procyonis* mtDNA. Therefore, more reliable, or at least more informative, markers should be considered for future work, especially for diagnostics/detection involving cross contamination or other *Baylisascaris* species. From the analysis in the present study, compared with the *cox2* gene, it seemed that *cytb* and *nad4* may be more suitable as molecular genetic markers for diagnosis and identification between *B. procyonis* and other related ascaridoids because of their higher variability. As shown in sliding window analysis (Figure 4), both the *cytb* and *nad4* genes were found to possess more variable positions per unit length of gene than *cox2*. Perhaps these markers can be further validated when additional Ascaridida mt genomes become available, especially from the genus *Baylisascaris*.

### Prediction of novel mt fragments for PCR specific identification within Ascaridida

Considering the level of nucleotide variability and nucleotide and/or amino acid sequence similarity within and between mt genes among the available Ascaridida mtDNAs, the genes *rrn*L and *cytb* were selected as potentially new mt fragments for PCR specific identification within the Ascaridida species. Subsequently, PCR primer pairs were designed targeting a 200 bp fragment in *rrn*L and a 548 bp fragment in *cytb* after manual inspection of the pre-aligned *rrn*L and *cytb* sequences. The primer pairs were: A (*rrn*L) forward 5′-GAGAACTGGCGGGG-3′, reverse 5′-CTCACACTGACTTACACACC-3′; B (*cytb*) forward 5′-TCCTTAGTAATGAGTATTGCGT-3′, reverse 5′-TATAACGACATTTGAAAAACACC-3′. The specificities of the two primer pairs designed herein were tested by PCR of the mt DNA from *B. procyonis*, *T. canis*, *T. leonina*, *A. suum* and the four congeners (*B. schroederi*, *B. ailuri*, *B. transfuga* and *B. columnaris*). As expected, the two targeted PCR amplification bands (200 bp *rrn*L and 548 bp *cytb*) were observed only in *B. procyonis* (data not shown). The PCR results demonstrated that the two fragments could be readily used to differentiate *B. procyonis* from other related ascaridoids, including the morphologically similar *Baylisascaris* spp. This finding would not only fill the technical gap by providing new mt fragments for effectively and specifically distinguishing between *B. procyonis* and *B. columnaris* [2], but it would also be useful for improving the efficiency and accuracy of environmental investigations for *B. procyonis*. Samples of *B. procyonis* eggs collected in nature are often cross-contaminated with eggs of other congeners, such as *B. transfuga* (from bears), *B. columnaris* (from skunks) and *B. melis* (from badgers), due to the presence of various infected hosts in an area. Such conditions could make it time-consuming or difficult for accurate identification of the parasitic species based on either the current methods of morphological examination [11] or existing molecular beacons (e.g., *cox2*) [2], [16]. Herein, we postulated that PCR amplification of the pair of the newly identified mt fragments (200 bp *rrn*L and 548 bp *cytb*) using primers developed in this study could rapidly and accurately identify and discriminate between *B. procyonis* and *B. transfuga*, *B. columnaris* and *B. melis*. However, other faint bands were also detected in our PCR assays, such as a 300bp band in *B. procyonis* and multiple bands in *B. transfuga* (not shown), which may relate to the specificity of primer pair A. These results suggested that the limited number of available mt genomes in the genus *Baylisascaris* is still a major barrier for screening effective mt fragments for PCR specific amplification used for identification and differentiation between *B. procyonis* and other congeners.

### Phylogeny

**Figure 5:**
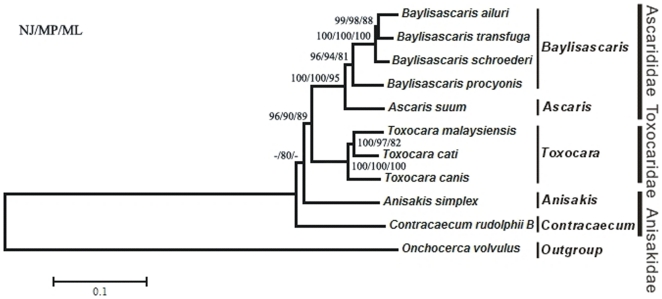
Phylogenetic relationships of ten Ascaridida species for which complete mtDNAs are available, inferred from NJ, MP and ML analysis for amino acid sequence data derived from 12 protein-coding genes, utilizing one filarioid species (*O. volvulus*) as the outgroup. Numbers above the branches represent bootstrap values derived from different analyses in the order: NJ/MP/ML. The scale indicates an estimate of substitutions per site, using the optimized model setting. The branches that were not universally supported with values of ≤50% are indicated with “-” in each supporting values of the node.

The availability of the *B. procyonis* mt genome provided us with another opportunity to probe the phylogenetic positions of the species *B. procyonis* within the genus *Baylisasaris* and of the genus *Baylisascaris* within the order Ascaridida. As expected from and congruent with previous phylogenetic analyses [23], [28], [29], [31], [38], phylogenies in this study inferred from the concatenated amino acid sequence dataset derived from 12 protein-coding mitochondrial genes. After the final alignment, the concatenated amino acid sequences (containing 3423 residues, including 2151 variable and 662 parsimony-informative) of 12 protein-coding genes for the 11 taxas (*B. procyonis B. schroederi*, *B. ailuri*, *B. transfuga*, *A. suum*, *T. canis*, *T. cati*, *T. malaysiensis*, *A. simplex*, *C. rudolphii* B and *Onchocerca volvulus*) were used to reconstruct the phylogenetic relationships based on maximum parsimony (MP), neighbor-joining (NJ) and maximum likelihood (ML) methods (Figure 5). All three phylogenetic analyses (NJ/MP/ML) conducted clearly supported the distinct classification positions of the genera *Baylisascaris* and *Ascaris* (family Ascarididae), *Toxocara* (family Toxocaridae), *Anisakis* (family Anisakidae) in the order Ascaridida, each as a monophyletic group with high statistical support (all bootstrap values >80) (Figure 5). Among the genus *Baylisascaris*, the phylogenetic analysis indicated a closer relationship between *B. procyonis* and *B. schroederi* than between *B. procyonis* and *B. ailuri* or *B. transfuga*. This finding was congruent with the results of a previous study using partial sequences of nuclear internal transcribed spacers (ITS) as genetic markers [42] and further confirmed the phylogenetic position of *B. procyonis* within the genus *Baylisascaris*. For the interrelationships of *B. ailuri*, *B. transfuga* and *B. schroederi* in the genus *Baylisascaris* and *T. canis*, *T. cati* and *T. malaysiensis* in genus *Toxocara*, their phylogenetic topologies were consistent with previously proposed molecular phylogeny based on mt data [23], [28].

In addition, the genus *Baylisascaris* was determined to be more closely related to *Ascaris* than to *Toxocara*, *Anisakis* and *Contracaecum* in the order Ascaridida in our phylogenetic analysis (Figure 5), which was consistent with results of previous morphological and molecular studies [23], [42]–[44]. But relationships between any of the *Baylisascaris* spp. (*B. procyonis B. schroederi*, *B. ailuri* and *B. transfuga*), *Toxocara* spp. (*T. canis*, *T. cati* and *T. malaysiensis*), *Ascaris* spp. (*A. suum*) and *Anisakis* spp. (*A. simplex*) and *Contracaecum* spp. (*C. rudolphii* B) were poorly inferred in the NJ and ML analyses (Figure 5). Therefore, a larger study of the evolutionary relationships among taxa within the order Ascaridida using mt data is warranted. The recent validation of the high-throughput sequencing technique for the sequencing of mt genomes provides a platform for an in-depth phylogenetic analysis of the order Ascaridida [32].

In conclusion, the complete mt genome of *B. procyonis* involved in 98–99% of *Baylisascaris* NLM and OLM cases in humans or other animals [11] was reported in our study. *B. procyonis* mtDNA showed a typical chromadorean mitogenome structure, but a long AT-rich region and high number of intergenic spacers made it unique compared with other nematodes characterized to date. In addition, the entire genome displayed notable levels of AT and GC skewing. Based on pairwise comparison and sliding window analysis within and between mt genes among the available 11 Ascaridida mtDNAs (including *B. procyonis* mtDNA), two new mt fragments (200 bp *rrn*L and 548 bp *cytb*) proved to be suitable as molecular targets for PCR based diagnosis and identification of *B. procyonis*. Finally the analysis of amino acids deduced from mtDNAs provided substantial support for the phylogenetic relationships of Ascaridida species; *B. procyonis* was more closely related to *B. schroederi* than to *B. ailuri* and *B. transfuga* in the genus *Baylisascaris*, and the genus *Baylisascaris* was more closely related to *Ascaris* than to *Toxocara*, *Anisakis* and *Contracaecum* in the order Ascaridida. The complete mitogenome of *B. procyonis* sequenced here is expected to render implications for molecular diagnostic methods, epidemiological investigations and ecological studies of *B. procyonis* and other related ascaridoids. The findings are important in the refinement of the phylogenetic relationships within the order Ascaridida and in accumulating valid markers for systematic, population genetic and evolutionary biological studies of parasitic nematodes of socio-economic importance.

## Materials and Methods

An adult female specimen of *B. procyonis* was obtained from an infected raccoon housed in the Chengdu Zoological Garden, Sichuan Province of China, after treatment with pyrantel pamoate. After washing in physiological saline, the morphological identification of the worm was performed based on the taxonomic key of Hartwich (1962). Total genomic DNA was isolated from a small portion (1 cm) of the specimen using the Universal Genomic DNA Extraction Kit Ver. 3.0 (TaKaRa, Japan). In order to further verify the identity of the specimen, the ITS1 and ITS2 sequences of nuclear ribosomal DNA (rDNA) were amplified by the PCR and compared with those previously reported for *B. procyonis* (Accession numbers: AB053230 and AB051231) [45].

The entire mt genome was amplified in ten overlapping segments (ranging in length from 873 bp to 2.47 kb) by PCR with Ex Taq Polymerase (TaKaRa, Japan), using ∼15 ng of total genomic DNA from the sample as template. The PCR primers were designed based on the alignments of the relatively conserved regions of congeneric *B. schroederi*, *B. ailuri* and *B. transfuga* and *A. suum* mt genome sequences. The names and corresponding primer sequences are shown in Table S4. All PCR reactions were carried out in a final reaction volume of 25 µl containing 1.5 µl of genomic DNA extract, 1 U Ex Taq Polymerase, 10× Ex Taq buffer, 0.2 mM of each dNTP, 10 pmol of each primer and ddH_2_O. PCR cycling conditions carried out in a Mastercycler Gradient 5331 thermocycler (Ependorf, Germany) were 5 min denaturation at 95°C, followed by 35 cycles of 30 s at 95°C, 30 s at 55°C and 2 to 5 min at 68°C according to the product length, with a final extension at 68°C for 10 min. Each PCR yielded a single band, detected in a 1% (W/V) agarose gel stained with ethidium bromide (not shown). Each amplicon was then purified using the TIANgel Midi Purification Kit (TiangenBiotech, China) and was subjected to automated sequencing, either directly or following sub-cloning into the pMD19-T vector (TaKaRa, Japan). To ensure maximum accuracy, each amplicon was sequenced twice independently, and in case of discrepancies a third PCR product was sequenced. Sequencing was performed using terminator-based cycle sequencing with BigDye chemistry (Applied Biosystems, Foster City, CA, USA) on an ABI 3730 DNA sequencer (Applied Biosystems), utilizing a primer-walking strategy (in both directions). The consensus sequences were assembled manually in a single contig and aligned with the published complete mt genome sequences of *B. transfuga* and *A. suum* [23], [27] using the Clustal X program, and the circular map was drawn using the program MacVector v. 9.5 (http://www.macvector.com/index.html). Genome annotation, and the comparisons with congeneric *B. schroederi*, *B. ailuri* and *B. transfuga* as well as other nematodes in the same order were performed using DNAMAN version 3.0 (Lynnon Biosoft, Quebec, Canada) and on-line blast tools available through the NCBI website [46]. Base composition and codon usage were calculated in DNAStar software (DNAStar, USA). Secondary structures of tRNA and rRNA were predicted using standard approaches [29]. The complete nucleotide sequences of mtDNAs for 11 Ascaridata species (including *B. procyonis*) were aligned using the MEGA 3.1 [47]. Subsequently, the complete alignment was used to accomplish sliding window analyses with the DnaSP ver.5.10 software package (http://www.ub.es/dnasp) [48]. A sliding window of 200 bp and steps of 20 bp were used to estimate nucleotide diversity Pi (π) for the complete alignment. Nucleotide diversity for the complete alignment was plotted against midpoint positions of each window, and gene boundaries were indicated. Based on Pi (π) values and nucleotide and/or amino acid sequence similarities within and between mt genes among the available Ascaridida mtDNAs, new mt fragments for PCR specific identification were selected, and corresponding PCR primer pairs for amplification were developed with either Primer Premier version 5.0 (Premier Biosoft International, Palo Alto, CA) or an on-line program PRIMER3 (www.genome.wi.mit.edu/cgi-bin/primer/primer3www.cgi), with the parameters modified as follows: melting temperature  = 45.0∼55.0°C, minimum number of 3′-end matches  = 3, optimal primer length interval  = (14, 24 bp), optimal PCR product length interval  =  (200, 400, 600 bp), minimum product length  = 150 bp. The specificities of the primer pairs designed were tested by PCR. All PCR reactions containing 10∼20 ng of the genomic DNA were performed in 50 µl volumes with 10 pmol of each primer, 250 µM of each dNTP, 2.0 mM MgCl_2_, and 2 U Taq polymerase under the following conditions: an initial denaturation at 94°C for 4 min followed by 35 cycles of 94°C for 30 sec, 50∼55°C for 30 sec, 72°C for 45 sec, and a final step at 72°C for 10 min.

For the phylogenetic analysis, in addition to *B. procyonis* mt genome sequenced in this study [GenBank: JF951366], the following mtDNAs from Nematoda were retrieved from GenBank: *B. ailuri* [GenBank: HQ671080], *B. transfuga* [GenBank: HQ671079], *B. schroederi* [GenBank: HQ671081], *A. suum* [GenBank: NC_001327], *T. canis* [GenBank: NC_010690], *T. cati* [GenBank: NC_010773], *T. malaysiensis* [GenBank: NC_010527], *A. simplex* [GenBank: NC_007934], *C. rudolphii* B [GenBank: NC_014870] and *O. volvulus* [GenBank: NC_001861]. Phylogenetic analyses were performed using the ten ascaridoid species (*B. procyonis*, *B. schroederi*, *B. ailuri*, *B. transfuga*, *T. canis*, *T. cati*, *T. malaysiensis*, *A. suum*, *A. simplex* and *C. rudolphii* B) as ingroups, and one filarioid species (*O. volvulus*) serving as outgroup. Twelve mitochondrial protein sequences were inferred using the Invertebrate Mitochondrial Code (Table five GenBank; http://www.ncbi.nlm.nih.gov/Taxonomy/Utils/wprintgc.cgi?mode=c#SG5). The predicted amino acid sequences were aligned using T-COFFEE 7.81 [49], the ambiguous regions within these alignments filtered with GBLOCKS 0.91 b [50], and then the filtered individual sequences were concatenated for subsequent phylogenetic analysis. The Dayhoff matrix model determined by ProtTest 2.0 [51] was employed in the NJ analysis using MEGA 3.1 [47]. MP phylogenetic reconstructions were conducted in PAUP* 4.0b10 [52], using heuristic searches with a tree-bisection-reconnection (TBR) branch-swapping algorithm and 1000 random-addition sequence replicates with ten trees held at each step, and finally the optimal topology was obtained using Kishino-Hasegawa. The ML computations were performed using PHYML 3.0 [53] under the LG +C4 + F + I model of amino acid substitution selected with ProtTest program. Branch supports were evaluated by bootstrapping analysis of 1000 replicates for NJ and MP trees, and 100 replicates for the ML tree.

## Supporting Information

Figure S1 **Predicted secondary structure of 22 tRNAs genes in the mt genome of** ***B. procyonis*** **.**
(TIF)

Figure S2 **Secondary structure predicted for the AT-rich region in the** ***B. procyonis*** **mt genome.**
(TIF)

Table S1 **Mitochondrial genome profiles of** ***B. procyonis*** **.**
(DOC)

Table S2 **Nucleotide codon usage for 12 protein-coding genes of the mitochondrial genome of** ***B. procyonis*** **.** Total number of codons is 3429. ^a^ Total number in all open reading frames. *** Stop codon. RSCU, relative synonymous codon usage.
(DOC)

Table S3 **Comparison of mitochondrial protein and rRNA genes with those of other nematodes sequenced in Ascaridida. ^a^: From Jex et al. (2008).** GenBank accession no. EU730761. ^b^: From Li et al. (2008). GenBank accession no. NC_010690. Bp: *B. procyonis*, Bs: *B. schroederi*, Ba: *B. ailuri*, Bt: *B. transfuga*, Asu: *A. suum*, Asi: *A. simplex*, Cr: *C. rudolphii* B, Tcan: *T. canis*, Tcat: *T. cati*, Tmal: *T. malaysiensis*.
(DOC)

Table S4 **List of the ten primer pairs for PCR amplification and their positions in the mt genome of** ***B. procyonis.***
(DOC)
