# Supplementary figures and images for: The Mitochondrial Genome of Baylisascaris procyonis
Source: PLoS One. 2011 Oct 28;6(10):e27066. doi: 10.1371/journal.pone.0027066 (PMC3203944; doi:10.1371/journal.pone.0027066)

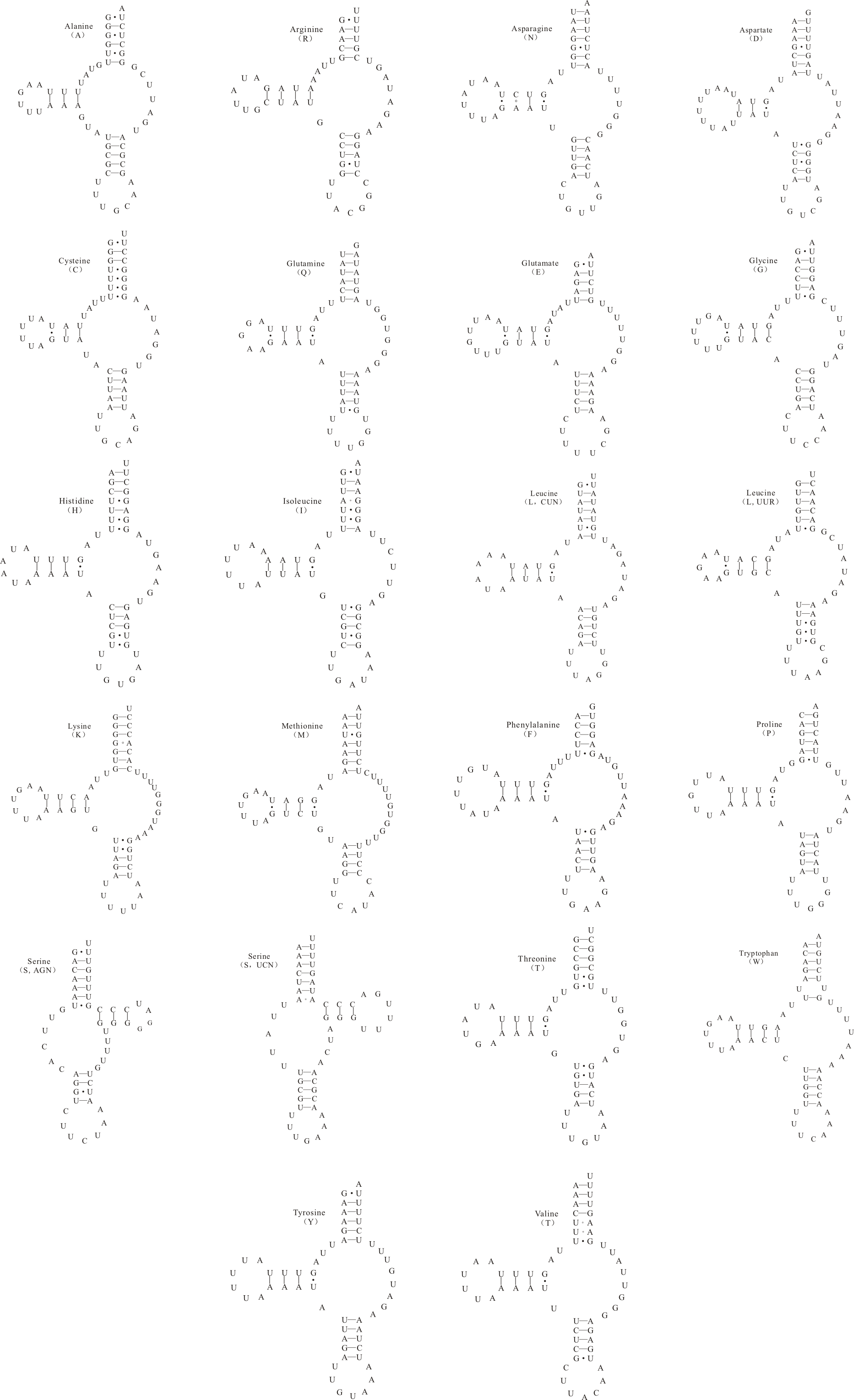

Supplement: Figure S1 — Predicted secondary structure of 22 tRNAs genes in the mt genome of B. procyonis . (TIF) [file pone.0027066.s001.tif]

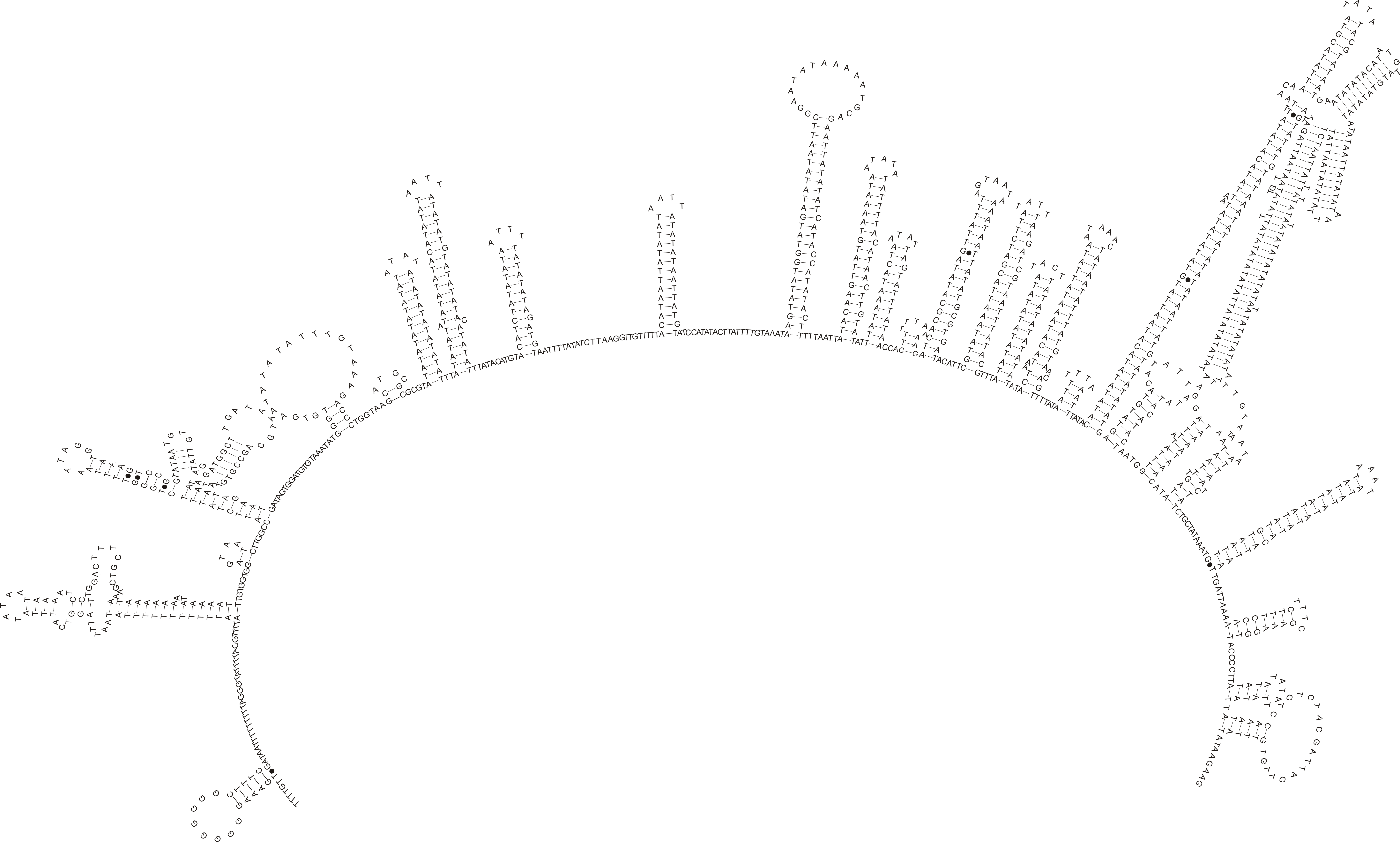

Supplement: Figure S2 — Secondary structure predicted for the AT-rich region in the B. procyonis mt genome. (TIF) [file pone.0027066.s002.tif]
