# Supplementary material for: The Mitochondrial Genome of Baylisascaris procyonis
Source: PLoS One. 2011 Oct 28;6(10):e27066. doi: 10.1371/journal.pone.0027066 (PMC3203944; doi:10.1371/journal.pone.0027066)
Supplement: Table S1 — Mitochondrial genome profiles of B. procyonis . (DOC) [file pone.0027066.s003.doc]

**Table S1. Mitochondrial genome profiles of *B. procyonis*.**

| **Gene/region** | **Positions** |  | **Sizes** | | |  | **Codons** | | **Anticodons** | **Intergenic  sequence** |
| --- | --- | --- | --- | --- | --- | --- | --- | --- | --- | --- |
|  |  |  | **No. of nt** |  | **No. of aa** |  | **Initiation** | **Termination** |  |  |
| ***nad*4L** | 1-234 |  | 234 |  | 77 |  | ATT | TAA |  | 2 |
| ***trn*W** | 235-291 |  | 57 |  |  |  |  |  | TCA | 0 |
| ***trn*E** | 292-348 |  | 57 |  |  |  |  |  | TTC | 1 |
| ***rrn*S** | 350-1049 |  | 700 |  |  |  |  |  |  | 19 |
| ***trn*S(UCN)** | 1069-1122 |  | 54 |  |  |  |  |  | TGA | 0 |
| **AT region** | 1123-2497 |  | 1375 |  |  |  |  |  |  | 0 |
| ***trn*N** | 2498-2555 |  | 58 |  |  |  |  |  | GTT | 0 |
| ***trn*Y** | 2576-2631 |  | 56 |  |  |  |  |  | GTA | 20 |
| ***nad*1** | 2632-3504 |  | 873 |  | 290 |  | TTG | TAG |  | 0 |
| ***atp*6** | 3510-4109 |  | 600 |  | 199 |  | ATA | TAG |  | 5 |
| ***trn*K** | 4113-4174 |  | 62 |  |  |  |  |  | TTT | 3 |
| ***trn*L(UUR)** | 4176-4230 |  | 55 |  |  |  |  |  | TAA | 1 |
| ***trn*S(AGN)** | 4231-4281 |  | 51 |  |  |  |  |  | TCT | 0 |
| **nad2** | 4282-5125 |  | 844 |  | 281 |  | GTG | T |  | 0 |
| ***trn*I** | 5126-5182 |  | 57 |  |  |  |  |  | GAT | 0 |
| ***trn*R** | 5186-5240 |  | 55 |  |  |  |  |  | ACG | 3 |
| ***trn*Q** | 5242-5296 |  | 55 |  |  |  |  |  | TTG | 1 |
| ***trn*F** | 5304-5364 |  | 61 |  |  |  |  |  | GAA | 7 |
| ***cytb*** | 5362-6468 |  | 1107 |  | 368 |  | GTG | TAG |  | -3 |
| ***trn*L(CUN)** | 6478-6534 |  | 57 |  |  |  |  |  | TAG | 9 |
| ***cox*3** | 6535-7302 |  | 768 |  | 255 |  | GTG | TAG |  | 0 |
| ***trn*T** | 7310-7364 |  | 55 |  |  |  |  |  | TGT | 7 |
| ***nad*4** | 7365-8594 |  | 1230 |  | 409 |  | TTG | TAG |  | 0 |
| **LNR region** | 8595-8712 |  | 118 |  |  |  |  |  |  | 0 |
| ***cox*1** | 8713-10,290 |  | 1578 |  | 525 |  | TTG | TAG |  | 0 |
| ***trn*C** | 10,290-10,345 |  | 56 |  |  |  |  |  | GCA | -1 |
| ***trn*M** | 10,355-10,414 |  | 60 |  |  |  |  |  | CAT | 9 |
| ***trn*D** | 10,415-10,473 |  | 59 |  |  |  |  |  | GTC | 0 |
| ***trn*G** | 10,476-10,531 |  | 56 |  |  |  |  |  | TCC | 2 |
| ***cox*2** | 10,532-11,230 |  | 699 |  | 232 |  | TTG | TAG |  | 0 |
| ***trn*H** | 11,234-11,289 |  | 56 |  |  |  |  |  | GTG | 3 |
| ***rrn*L** | 11,290-12,253 |  | 964 |  |  |  |  |  |  | 0 |
| ***nad*3** | 12,254-12,589 |  | 336 |  | 111 |  | TTG | TAA |  | 0 |
| ***nad*5** | 12,590-14,174 |  | 1585 |  | 528 |  | ATT | T |  | 0 |
| ***trn*A** | 14,175-14,230 |  | 56 |  |  |  |  |  | TGC | 0 |
| ***trn*P** | 14,231-14,286 |  | 56 |  |  |  |  |  | TGG | 0 |
| ***trn*V** | 14,288-14,344 |  | 57 |  |  |  |  |  | TAC | 1 |
| ***nad*6** | 14,345-14,779 |  | 435 |  | 144 |  | TTG | TAG |  | 0 |
