# Supplementary material for: The Mitochondrial Genome of Baylisascaris procyonis
Source: PLoS One. 2011 Oct 28;6(10):e27066. doi: 10.1371/journal.pone.0027066 (PMC3203944; doi:10.1371/journal.pone.0027066)
Supplement: Table S2 — Nucleotide codon usage for 12 protein-coding genes of the mitochondrial genome of B. procyonis . Total number of codons is 3429. a Total number in all open reading frames. *** Stop codon. RSCU, relative synonymous codon usage. (DOC) [file pone.0027066.s004.doc]

**Table S2. Nucleotide codon usage for 12 protein-coding genes of the mitochondrial genome of *B. procyonis*.**

| **AA** | **Codon** | **No.^a^** | **%** | **RSCU** | **AA** | **Codon** | **No.^a^** | **%** | **RSCU** | **AA** | **Codon** | **No.^a^** | **%** | **RSCU** | **AA** | **Codon** | **No.^a^** | **%** | **RSCU** |
| --- | --- | --- | --- | --- | --- | --- | --- | --- | --- | --- | --- | --- | --- | --- | --- | --- | --- | --- | --- |
| **Phe** | **TTT** | 478 | 13.9 | 1.955 | **Ser** | **TCT** | 142 | 4.1 | 3.528 | **Ala** | **GCT** | 83 | 2.4 | 3.162 | **Asp** | **GAT** | 70 | 2.0 | 1.842 |
|  | **TTC** | 11 | 0.3 | 0.045 |  | **TCC** | 1 | <0.1 | 0.025 |  | **GCC** | 11 | 0.3 | 0.419 |  | **GAC** | 6 | 0.2 | 0.158 |
| **Leu** | **TTA** | 106 | 3.1 | 0.491 |  | **TCA** | 11 | 0.3 | 0.273 |  | **GCA** | 3 | 0.1 | 0.114 | **Glu** | **GAA** | 16 | 0.5 | 0.421 |
|  | **TTG** | 326 | 9.5 | 1.509 |  | **TCG** | 7 | 0.2 | 0.174 |  | **GCG** | 8 | 0.2 | 0.305 |  | **GAG** | 60 | 1.7 | 1.579 |
| **Leu** | **CTT** | 57 | 1.7 | 3.040 | **Ser** | **AGT** | 119 | 3.5 | 2.404 | **Tyr** | **TAT** | 155 | 4.5 | 1.834 | **Cys** | **TGT** | 55 | 1.6 | 1.896 |
|  | **CTC** | 1 | <0.1 | 0.053 |  | **AGC** | 6 | 0.2 | 0.121 |  | **TAC** | 14 | 0.4 | 0.166 |  | **TGC** | 3 | 0.1 | 0.103 |
|  | **CTA** | 3 | 0.1 | 0.154 |  | **AGA** | 26 | 0.7 | 0.525 | ******* | **TAA** | 2 | <0.1 | 0.400 | **Trp** | **TGA** | 15 | 0.4 | 0.400 |
|  | **CTG** | 14 | 0.4 | 0.747 |  | **AGG** | 47 | 1.4 | 0.949 | ******* | **TAG** | 8 | 0.2 | 1.600 |  | **TGG** | 60 | 1.7 | 1.600 |
| **Ile** | **ATT** | 192 | 5.6 | 1.920 | **Pro** | **CCT** | 69 | 2.0 | 3.325 | **His** | **CAT** | 54 | 1.6 | 1.895 | **Arg** | **CGT** | 28 | 0.8 | 3.200 |
|  | **ATC** | 8 | 0.2 | 0.080 |  | **CCC** | 4 | 0.1 | 0.193 |  | **CAC** | 3 | 0.1 | 0.105 |  | **CGC** | 2 | <0.1 | 0.228 |
| **Met** | **ATA** | 42 | 1.2 | 0.488 |  | **CCA** | 3 | 0.1 | 0.144 | **Gln** | **CAA** | 7 | 0.2 | 0.333 |  | **CGA** | 2 | <0.1 | 0.228 |
|  | **ATG** | 130 | 3.8 | 1.512 |  | **CCG** | 7 | 0.2 | 0.337 |  | **CAG** | 35 | 1.0 | 1.667 |  | **CGG** | 3 | 0.1 | 0.343 |
| **Val** | **GTT** | 241 | 7.0 | 2.465 | **Thr** | **ACT** | 90 | 2.6 | 3.273 | **Asn** | **AAT** | 103 | 3.0 | 1.890 | **Gly** | **GGT** | 135 | 3.9 | 2.560 |
|  | **GTC** | 19 | 0.5 | 0.194 |  | **ACC** | 3 | 0.1 | 0.109 |  | **AAC** | 6 | 0.2 | 0.110 |  | **GGC** | 6 | 0.2 | 0.114 |
|  | **GTA** | 37 | 1.1 | 0.378 |  | **ACA** | 5 | 0.1 | 0.182 | **Lys** | **AAA** | 16 | 0.5 | 0.337 |  | **GGA** | 19 | 0.5 | 0.360 |
|  | **GTG** | 94 | 2.7 | 0.962 |  | **ACG** | 12 | 0.3 | 0.436 |  | **AAG** | 79 | 2.3 | 1.663 |  | **GGG** | 51 | 1.5 | 0.967 |

Total number of codon is 3429.

^a^ Total number in all open reading frames.

******* Stop codon.

RSCU refers to relative synonymous codon usage.
