# Supplementary material for: The Mitochondrial Genome of Baylisascaris procyonis
Source: PLoS One. 2011 Oct 28;6(10):e27066. doi: 10.1371/journal.pone.0027066 (PMC3203944; doi:10.1371/journal.pone.0027066)
Supplement: Table S3 — Comparison of mitochondrial protein and rRNA genes with those of other nematodes sequenced in Ascaridida. a: From Jex et al. (2008). GenBank accession no. EU730761. b: From Li et al. (2008). GenBank accession no. NC_010690. Bp: B. procyonis, Bs: B. schroederi, Ba: B. ailuri, Bt: B. transfuga, Asu: A. suum, Asi: A. simplex, Cr: C. rudolphii B, Tcan: T. canis, Tcat: T. cati, Tmal: T. malaysiensis. (DOC) [file pone.0027066.s005.doc]

**Table S3. Comparison of mitochondrial protein and rRNA genes with those of other nematodes** sequenced in Ascaridida.

|  | **Sizes of mitochondrial genes** | | | | | | | | | | |  | **% nucleotide/amino acid identity** | | | | | | | | | |
| --- | --- | --- | --- | --- | --- | --- | --- | --- | --- | --- | --- | --- | --- | --- | --- | --- | --- | --- | --- | --- | --- | --- |
|  | 1 | 2 | 3 | 4 | 5 | 6 | 7 | 8 | 9 | 10 | 11 |  |  | | | | | | | | | |
|  | Bp | Bs | Ba | Bt | Asu | Asi | Cr | Tcan^a^ | Tcan^b^ | Tcat | Tmal |  | 1 vs 2 | 1 vs 3 | 1 vs 4 | 1 vs 5 | 1 vs 6 | 1 vs 7 | 1 vs 8 | 1 vs 9 | 1 vs 10 | 1 vs 11 |
| **Protein genes** |  |  |  |  |  |  |  |  |  |  |  |  |  |  |  |  |  |  |  |  |  |  |
| ***atp6*** | 600 | 600 | 600 | 600 | 600 | 600 | 600 | 598 | 598 | 598 | 598 |  | 89.3(92.5) | 88.2(91.0) | 87.3(92.0) | 87.0(92.0) | 81.8(84.9) | 80.3(84.9) | 82.3(81.4) | 82.2(8.14) | 82.3(80.9) | 82.8(82.4) |
| ***cox1*** | 1578 | 1578 | 1575 | 1578 | 1578 | 1576 | 1576 | 1578 | 1578 | 1578 | 1581 |  | 91.8(96.6) | 91.5(89.9) | 92.2(96.4) | 92.1(97.9) | 85.2(91.4) | 84.7(93.1) | 88.0(94.7) | 87.8(94.9) | 89.1(95.4) | 88.5(94.7) |
| ***cox2*** | 699 | 699 | 699 | 699 | 699 | 699 | 696 | 714 | 714 | 711 | 711 |  | 90.8(98.3) | 91.4(98.3) | 91.7(98.3) | 91.7(96.5) | 84.7(90.1) | 83.5(92.7) | 85.7(91.6) | 85.6(91.6) | 83.3(91.9) | 86.8(91.1) |
| ***cox3*** | 768 | 768 | 768 | 768 | 768 | 766 | 766 | 768 | 768 | 768 | 768 |  | 88.9(93.7) | 88.0(93.3) | 88.0(83.9) | 89.4(94.1) | 81.8(87.4) | 82.8(89.0) | 84.1(91.0) | 84.0(91.0) | 81.4(90.2) | 83.3(89.4) |
| ***cytb*** | 1107 | 1107 | 1107 | 1107 | 1098 | 1099 | 1107 | 1107 | 1107 | 1107 | 1107 |  | 87.9(91.0) | 86.6(91.0) | 87.2(91.8) | 87.3(89.7) | 76.4(76.6) | 76.2(74.7) | 78.9(80.2) | 78.2(80.2) | 79.5(81.0) | 78.9(79.6) |
| ***nad1*** | 873 | 873 | 873 | 873 | 873 | 873 | 873 | 882 | 873 | 873 | 873 |  | 90.4(94.5) | 90.3(95.9) | 90.6(95.9) | 90.3(92.8) | 83.2(87.6) | 80.8(85.5) | 84.3(87.4) | 85.3(88.3) | 84.6(89.7) | 84.4(89.0) |
| ***nad2*** | 844 | 845 | 845 | 845 | 844 | 846 | 846 | 844 | 844 | 844 | 844 |  | 90.4(91.1) | 90.2(91.1) | 89.1(89.7) | 82.5(82.3) | 79.2(80.4) | 73.8(71.5) | 78.2(74.0) | 78.5(75.8) | 80.7(76.5) | 78.2(74.4) |
| ***nad3*** | 336 | 336 | 336 | 336 | 336 | 336 | 336 | 330 | 336 | 336 | 336 |  | 89.3(94.6) | 88.4(94.6) | 87.5(95.5) | 87.8(91.0) | 80.4(82.9) | 81.5(82.8) | 82.7(84.7) | 83.6(86.5) | 83.0(86.5) | 82.1(85.6) |
| ***nad4*** | 1230 | 1230 | 1230 | 1230 | 1230 | 1230 | 1230 | 1230 | 1230 | 1230 | 1230 |  | 87.1(93.1) | 87.2(93.1) | 87.6(93.4) | 86.0(91.9) | 76.7(80.7) | 75.2(76.5) | 78.0(84.1) | 78.0(83.6) | 77.6(83.6) | 77.6(82.6) |
| ***nad4L*** | 234 | 234 | 234 | 234 | 234 | 232 | 234 | 232 | 232 | 232 | 232 |  | 85.9(92.2) | 86.7(94.8) | 88.0(94.8) | 88.9(96.1) | 81.6(88.3) | 85.9(89.6) | 78.6(85.7) | 79.9(87.0) | 83.3(87.0) | 79.9(83.1) |
| ***nad5*** | 1585 | 1585 | 1585 | 1585 | 1585 | 1582 | 1582 | 1576 | 1582 | 1582 | 1582 |  | 87.6(89.0) | 86.9(89.6) | 87.4(90.0) | 83.4(86.6) | 79.6(81.1) | 78.8(79.4) | 81.2(83.7) | 81.4(83.9) | 81.7(85.4) | 82.4(84.8) |
| ***nad6*** | 435 | 435 | 435 | 435 | 435 | 435 | 435 | 435 | 434 | 434 | 434 |  | 87.8(92.4) | 86.9(88.9) | 87.1(88.9) | 86.2(90.3) | 77.7(79.9) | 75.6(73.6) | 79.1(75.7) | 78.8(75.7) | 79.8(79.1) | 80.5(78.5) |
| **rRNA genes** |  |  |  |  |  |  |  |  |  |  |  |  |  |  |  |  |  |  |  |  |  |  |
| ***rrnS*** | 700 | 694 | 695 | 694 | 701 | 699 | 689 | 693 | 697 | 696 | 696 |  | 92.3 | 92.6 | 91.5 | 91.0 | 81.9 | 78.1 | 80.4 | 81.1 | 80.1 | 79.6 |
| ***rrnL*** | 964 | 963 | 962 | 964 | 960 | 957 | 961 | 924 | 958 | 955 | 955 |  | 86.2 | 86.2 | 85.9 | 86.7 | 76.3 | 76.1 | 75.9 | 78.8 | 79.2 | 78.9 |

^a^: From Jex et al. (2008). GenBank accession no. EU730761.

^b^: From Li et al. (2008). GenBank accession no. NC_010690.

Bp: *B. procyonis*, Bs: *B. schroederi*, Ba: *B. ailuri*, Bt: *B. transfuga*, Asu: *A. suum*, Asi: *A. simplex*, Cr: *C. rudolphii B*, Tcan: *T. canis*, Tcat: *T. cati*, Tmal: *T. malaysiensis*.
