# Supplementary material for: The Mitochondrial Genome of Baylisascaris procyonis
Source: PLoS One. 2011 Oct 28;6(10):e27066. doi: 10.1371/journal.pone.0027066 (PMC3203944; doi:10.1371/journal.pone.0027066)
Supplement: Table S4 — List of the ten primer pairs for PCR amplification and their positions in the mt genome of B. procyonis. (DOC) [file pone.0027066.s006.doc]

**Table S4. List of the ten primer pairs for PCR amplification and their positions in the mt genome of *B. procyonis.***

| **Primer name** |  | **Location based  on *B. procyonis* mtDNA** |  | **Primer sequence ( 5' to 3')** |
| --- | --- | --- | --- | --- |
| **BP-1** |  | **13977-13999 907-929** |  | **Forward: AGTTTTTGGTTGATTTTTTTGCT Reverse: CTGTCTTTTACATTTTCAACTTC** |
| **BP-2** |  | **700-726 3146-3169** |  | **Forward: GTTCTTAAATTATCTTTGGAGGTTGAG Reverse: GAAAAGGTAAATAAATAACAAACA** |
| **BP-3** |  | **2536-2560 4680-4703** |  | **Forward: TCAACGGGTTTTACTCTTAAGATAT Reverse: ACAAACAGCTGAAAATAGCAAACA** |
| **BP-4** |  | **4157-4179 5463-5487** |  | **Forward: AATGGGTTTTCACACCCTGGTTG Reverse: TGTTAAAATCTGGAACCCCAAAACC** |
| **BP-5** |  | **4929-4950 6706-6727** |  | **Forward: TAAGATTTTTGTTTTGAGTGAG Reverse: CCCTAAGACCTTCTATTACAAT** |
| **BP-6** |  | **6333-6355 8356-8380** |  | **Forward: AGTTTTGAGTTGGCTTGGGCAGT Reverse: TCAGACACAAAAGACATTGACGGTG** |
| **BP-7** |  | **8069-8093 10201-10222** |  | **Forward: TTGTTGTATGTTTCAGAGTGACGCG Reverse: TAAGGCTATATTCAGGCCCGCT** |
| **BP-8** |  | **10018-10040 12442-12467** |  | **Forward: CATTTTGCCAGTATTCATGGTTA Reverse: TTACTACCTCCAAGTCAAAAATAACA** |
| **BP-9** |  | **12078-12097 12926-12950** |  | **Forward: GGGAGGAGAGGACTTTGGGT Reverse: TATAAAACAAAACCAAAAAAAACCT** |
| **BP-10** |  | **12747-12771 14757-14781** |  | **Forward: TTATTTTGCTTTTGGTTACTGTAAG Reverse: CACTAAACTTTACGTAAAGCACCCG** |
